# Theoretical Study of the Local Surface Plasmon Resonance Properties of Silver Nanosphere Clusters

**DOI:** 10.1007/s11468-013-9541-y

**Published:** 2013-04-20

**Authors:** Ye-Wan Ma, Zhao-Wang Wu, Li-Hua Zhang, Jie Zhang, Guo-Shu Jian, Shi Pan

**Affiliations:** 1 School of Physics and Electronic Engineering, Anqing Normal University, Anqing, 246011 China; 2 School of Physics and Optoelectronics Technology, Dalian University of Technology, Dalian, 116024 China

**Keywords:** Local surface plasmon resonance, Silver nanosphere clusters, Green function, Optical efficiencies

## Abstract

The local surface plasmon resonance properties in systems consisting of silver nanosphere clusters are studied by Green’s function. The extinction, absorption, and scattering efficiencies band of two, three, and more silver nanospheres clusters are discussed in detail. The clusters show new types of the local surface plasmon resonances compared with single silver nanosphere. Our results suggest that the resonances depend strongly on individual particles’ characteristics such as their shapes, gap distances, directions and polarizations of incident light waves, and the number of clusters. The spectrum shows that equilateral triangle nanospheres has a good absorption peak, while the better red-shifted with three aligned nanospheres. In addition, the distributions of electric field intensity for three and four touched silver nanospheres are also investigated. The study is useful to broaden the application scope of Raman spectroscopy and nanooptics.

## Introduction

It is well known that the noble metal nanoparticles (silver (Ag), gold (Au), and copper (Cu)) have different optical, electromagnetic properties from bulk materials [1, 2] owing to quantum sizes and surface effects. Recently, a large amount of studies has been developed to study the optical properties of noble metal nanoparticles which could support local surface plasmon resonance (LSPR). LSPR are electromagnetic modes associated with the excitation of collective oscillations of the electronic charge density in metals. The oscillation frequency is determined by four factors: the density of electrons, the electron mass, the size, and the shape of the charge distribution. Many unique optical properties can be achieved when adjusting the structure, morphology size, and composition of the metal nanoparticles [3–7]. Consequently, manufacturing and application of metallic nanoparticles has become very active topics in materials science.

Furthermore, the strong optical field generated in these systems could be used in surface-enhanced Raman scattering and in devising new configuration for chemical and material science [4, 5]. As these devices are strongly sensitive to light frequency, it is interesting to dispose tunable nanoparticles to modify their frequency range. There are a fair number of experimental/theoretical/numerical studies devoted to this subject, for example, the groups of Halas and Schatz have studied some gold/sliver shapes such as nanosphere, nanoshell and triangular nanoprisms with discrete-dipole approximation (DDA) simulation and experiments [7–9]. Besides, other authors such as Pendry et al. have developed very original methods based on conformal or nonconformal transformations to describe the interaction between two particles [10]. In addition, clusters are also found in the literature on optical antennas [11]. Single- and two-nanoparticle spheres/cylinders were previously studied theoretically with Green’s function by Martin [12, 13]. In this paper, we report our study on the optical properties of two, three, and more silver spheres clusters using Green’s function. The silver permittivity data are cited from Johnson and Christy [14].

This paper is organized as follows. In Section “Green Function Method”, we briefly describe the numerical simulation method based on Green’s function. In Section “Numerical Simulation: Results and Analysis”, we present spectrum calculations in detail for two nanosphere with different incident directions and also its polarizations, its clusters, and the distribution of electrical field intensity. In Section “Conclusion”, we summarize our study.

## Green Function Method

Generally, some numerical simulation methods are used to study the optical properties of noble metal nanoparticles such as finite-difference time domain [15], finite element method [16], and DDA [17]. In this paper, Green’s function [18–20] is used to study the optical properties of silver nanoparticles, which is also called coupled dipole method [21]. First, let us briefly outline the main features of the theoretical scattering formalism with the Green’s function on which the numerical simulation is based and associated numerical methods. The scattering object with a dielectric function $\varepsilon _{s}(\textbf {\textit {r}},\omega )$ embedded in an infinitely homogenous background medium with a dielectric parameter of $\varepsilon _{m}(\textbf {\textit {r}},\omega )$ could be expressed as the following three-dimension vector Lippmann–Schwinger integral equation:

1 $$ \textbf{\textit{E}}(\textbf{\textit{r}},\omega)=\textbf{\textit{E}}_{inc}(\textbf{\textit{r}},\omega) +\int_{V'}\textbf{\textit{G}}_{0}(\textbf{\textit{r}},\textbf{\textit{r}}',\omega) \Delta\varepsilon(\textbf{\textit{r}}',\omega)\textbf{\textit{E}}(\textbf{\textit{r}}',\omega)d{\textit{V}}', $$

where $\textbf {\textit {G}}_{0}(\textbf {\textit {r}},\textbf {\textit {r}}',\omega )$ is the Green’s tensor for an infinitely homogeneous background medium, $\varepsilon _{m}$, and is expressed as:

2 $$\begin{array}{rll} \textbf{\textit{G}}_{0}(\textbf{\textit{r}},\textbf{\textit{r}}',\omega) &=&k^{2}_{0}\left(\textbf{\textit{I}}+\frac{ik_{m}R-1}{k^{2}_{m}R^{2}}\textbf{\textit{I}} +\frac{3-3ik_{m}R-k^{2}_{m}R^{2}}{k^{2}_{m}R^{4}}\textbf{\textit{R}}\textbf{\textit{R}}\right)\\ &&\times\frac{exp(ik_{m}R)}{4\pi R}, \qquad \textbf{\textit{r}}\neq \textbf{\textit{r}}',\qquad \ \end{array} $$

where $\textbf {\textit {I}}$ is the unit dyadic, $\textbf {\textit {R}}=\textbf {\textit {r}}-\textbf {\textit {r}}'$, $R=|\textbf {\textit {r}}-\textbf {\textit {r}}'|$, and $\Delta \varepsilon (\textbf {\textit {r}}',\omega )=\varepsilon _{s}-\varepsilon _{m}$. There is a singularity in Eq. 2 when $\textbf {\textit {r}}=\textbf {\textit {r}}'$, which have been solved by Yaghjian in detail [22]. The implicit (Eq. 1) can be solved via numerical simulation based on discretization, which results in:

3 $$\begin{array}{rll} \textbf{\textit{E}}(\textbf{\textit{r}},\omega)&=&\textbf{\textit{E}}_{inc}(\textbf{\textit{r}},\omega) +\sum\limits^{N}_{j=1}\textbf{\textit{G}}_{0}(\textbf{\textit{r}}_{i},\textbf{\textit{r}}_{j},\omega) \Delta\varepsilon_{j}\textbf{\textit{E}}(\textbf{\textit{r}}_{j},\omega)V_{j},\\ && i=1,\cdots, N \end{array} $$

where $V_{j}$ is the volume of the scattering particle. Since the scalar Green’s tensor is dependent only on the absolute relative distance $\textbf {\textit {R}}$ (Eq. 3), and is reciprocal, i.e., $\textbf {\textit {G}}(\textbf {\textit {r}}_{i},\textbf {\textit {r}}_{j}) =\textbf {\textit {G}}(\textbf {\textit {r}}_{j},\textbf {\textit {r}}_{i})$, or $\textbf {\textit {G}}(\textbf {\textit {r}}_{i},\textbf {\textit {r}}_{j})=\textbf {\textit {G}}_{i-j}$, we can generate the following equation by substituting $\textbf {\textit {G}}(\textbf {\textit {r}}_{i},\textbf {\textit {r}}_{j})=\textbf {\textit {G}}_{i-j}$ into Eq. 3 and rearranging terms:

4 $$\sum\limits^{N}_{j=1}(\textbf{\textit{I}}-\textbf{\textit{G}}_{i-j}\Delta\varepsilon_{j}V_{j}) \textbf{\textit{E}}_{j}=\textbf{\textit{E}}_{inc}(\textbf{\textit{r}}_{i},\omega),\quad\quad i=1,\cdots,N\\ $$

where $\textbf {\textit {E}}_{j}$ and $\textbf {\textit {E}}_{inc}$ are $3N$-dimensional vector, and $\textbf {\textit {G}}_{i-j}$ is a 3*N* × 3*N* matrix. The total electric field can be derived, along with spectral and optical parameters, after solving these $3N$ complex linear equations. The optical efficiencies [23] (i.e., extinction cross section, absorption cross section, and scattering cross section) are defined as:

5 $$\begin{array}{rll} C_{\mathrm{ext}}&=&\frac{4\pi k}{|\textbf{\textit{E}}_{inc}|^{2}}\sum^{N}_{i=1}Im\big(\textbf{\textit{E}}^{*}_{inc}\cdot\textbf{\textit{P}}_{i}\big), \\ C_{abs}&=&\frac{4\pi k}{|\textbf{\textit{E}}_{inc}|^{2}}\sum^{N}_{i=1}\left\{Im\big[\textbf{\textit{P}}_{i}\cdot(\alpha^{-1}_{i})^{*}\textbf{\textit{P}}^{*}_{i}\big] -\frac{2}{3}k^{3}|\textbf{\textit{P}}_{i}|^{2}\right\}, \end{array} $$

where $\textbf {\textit {P}}_{i}=\alpha _{i}\cdot \textbf {\textit {E}}_{loc}$, $\textbf {\textit {E}}_{loc}=[(\varepsilon _{s}+2\varepsilon _{m})/3]\textbf {\textit {E}}$ with $\alpha _{i}=\frac {(\varepsilon _{s}(\textbf {\textit {r}}_{i},\omega )-\varepsilon _{m}(\textbf {\textit {r}}_{i},\omega ))} {(\varepsilon _{s}(\textbf {\textit {r}}_{i},\omega )+2\varepsilon _{m}(\textbf {\textit {r}}_{i},\omega ))}\frac {3V}{4\pi }$ and scattering cross section can be obtained by $C_{sca}=C_{\mathrm {ext}}-C_{abs}$. The extinction efficiency and absorption efficiency are $Q_{\mathrm {ext}}=C_{\mathrm {ext}}/S$ and $Q_{sca}=C_{sca}/S$, where *S* is the effective area of scattering particles.

In order to achieve desired simulation accuracy, it is necessary to use a large number of dipoles, to model features properly with the Green’s function. Due to surface effects coming from a given cubic lattice bounded by a particle shape, Noguez [24] gave a formula to calculate the numbers of surface dipoles to surface effects. It means that we should choose more dipoles (e.g., 10^3^ or more) to model a geometry. In this paper, depending on the error in the calculation for silver particles, the cube size of each dipole is lesser than 1.5 nm, and the number of dipoles (N) is about 1.7 × 10^4^ for each sphere. For the linear (Eq. 4), we could solve them effectively with complex-conjugate gradient method [25] and fast Fourier transform algorithm [26, 27].

## Numerical Simulation: Results and Analysis

**Fig. 1:**
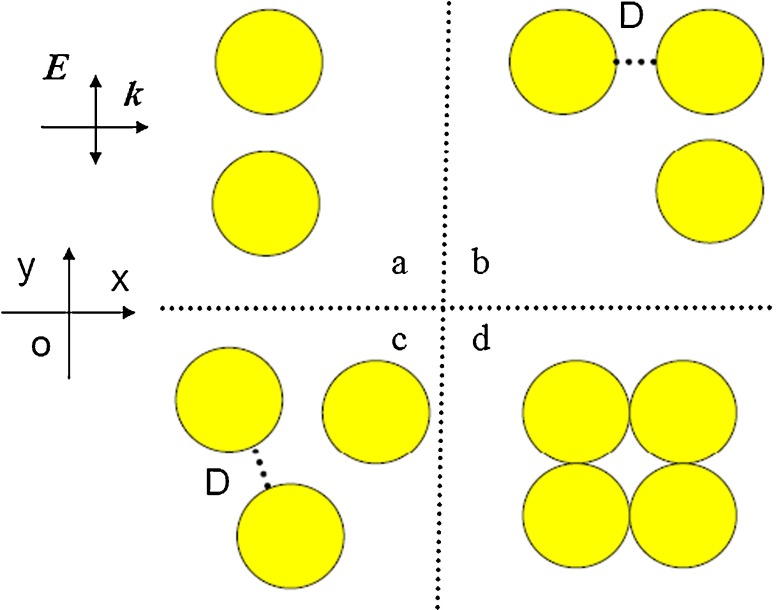
Geometry for two/three and more silver spheres clusters with radius and individual separation *r* and *D*, illuminated by plane wave with $\textbf {\textit {k}}$ propagating and $\textbf {\textit {E}}$ polarization direction

The numerical geometries are given in Fig. 1. Where *r* is the radius of silver nanosphere, and *D* denotes the gap distance between the adjacent silver nanospheres. $\textbf {\textit {k}}$ and $\textbf {\textit {E}}$ are the propagating direction and the electric field polarization of incident plane waves, respectively. Figure 1a denotes two identical silver nanospheres with the gap distance *D*, Fig. 1b shows right triangle shape with three identical silver nanospheres, Fig. 1c equilateral triangle shape, and Fig. 1d four touched nanospheres.

**Fig. 2:**
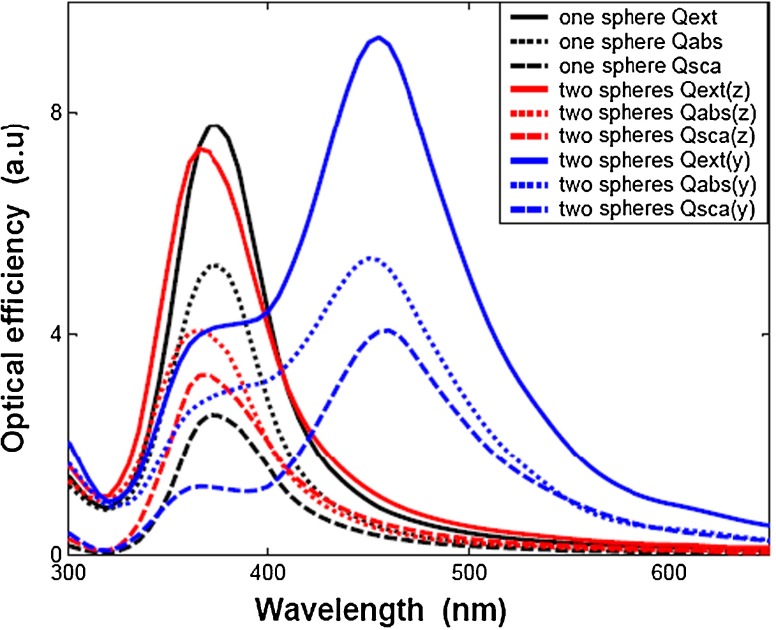
The extinction efficiencies of *one sphere* and *two spheres* with different direction polarizations (*y* and *z* direction)

First, the optical efficiencies (i.e., extinction efficiency, absorption efficiency, and scattering efficiency) are given for two silver spheres with 3-nm distance and one sphere by the wave propagating directions parallel to *x* direction, while the electric field polarization along the *y* direction and *z* direction, respectively, as shown in Fig. 1a, and the nanoparticles’ radius to 30 nm. The simulation results, as plotted in Fig. 2, show that the peak position of optical efficiencies for two silver nanospheres with *z* polarization are similar to optical efficiencies of one sphere with only one peak at about 370 nm, which just a smaller blue-shifted. The data also illustrate a local minimum for the extinction efficiencies at about 320 nm, where both the real and imaginary parts of Ag dielectric parameter are expected to reach zero. Its spectral feature is inherent to the Ag materials properties, independent to the particle’s geometries, sizes, which could be seen bellow [7, 28]. In addition, the main contribution of scattering and absorption efficiencies to the extinction efficiency can be seen clearly from Fig. 2. The absorption efficiency is the primary contributor to the extinction efficiency for $\lambda <400$ nm, which are associated with the plasmon resonances and inherent to the nanosphere geometry and polarization direction. However, both the scattering and absorption efficiencies play equal contribution to extinction efficiency for $\lambda >400$ nm and show a long tail. Compared with one nanosphere, the extinction efficiency and absorption efficiency for two nanospheres is smaller than one sphere, while the scattering efficiency is bigger than one sphere due to augmenting scattering volume. If we look only at extinction efficiency, it is impossible to observe such features since the scattering effects hide them. It is also interesting to find out that there are two peaks for two nanospheres by *y* polarization, one at about 370 nm and the other at about 470 nm. It also shows that the intensity of peak position at about 470 nm is greater than *z* polarization and one single nanosphere, and a significant increase in plasmon resonance width which probably comes from enhanced radiation damping due to the larger volume of two nanoparticles compared with one sphere, the same to three nanospheres seen below. The absorption efficiency is the primary contributor to the extinction efficiency for $\lambda <550$ nm. Compared with the absorption efficiency and scattering efficiency, it shows that there is a good absorption efficiency for silver nanosphere from Fig. 2.

**Fig. 3:**
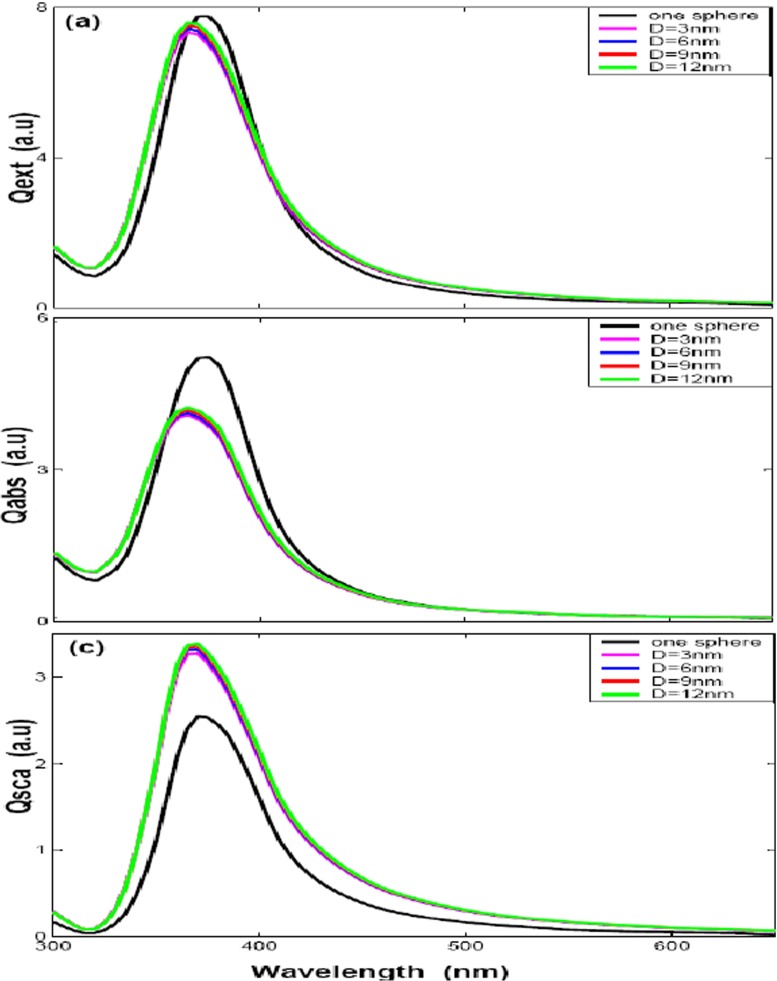
The optical efficiencies of *two spheres* with different gap distances (*D* = 0, 3, 6, 9, and 12 nm) by z direction polarization

**Fig. 4:**
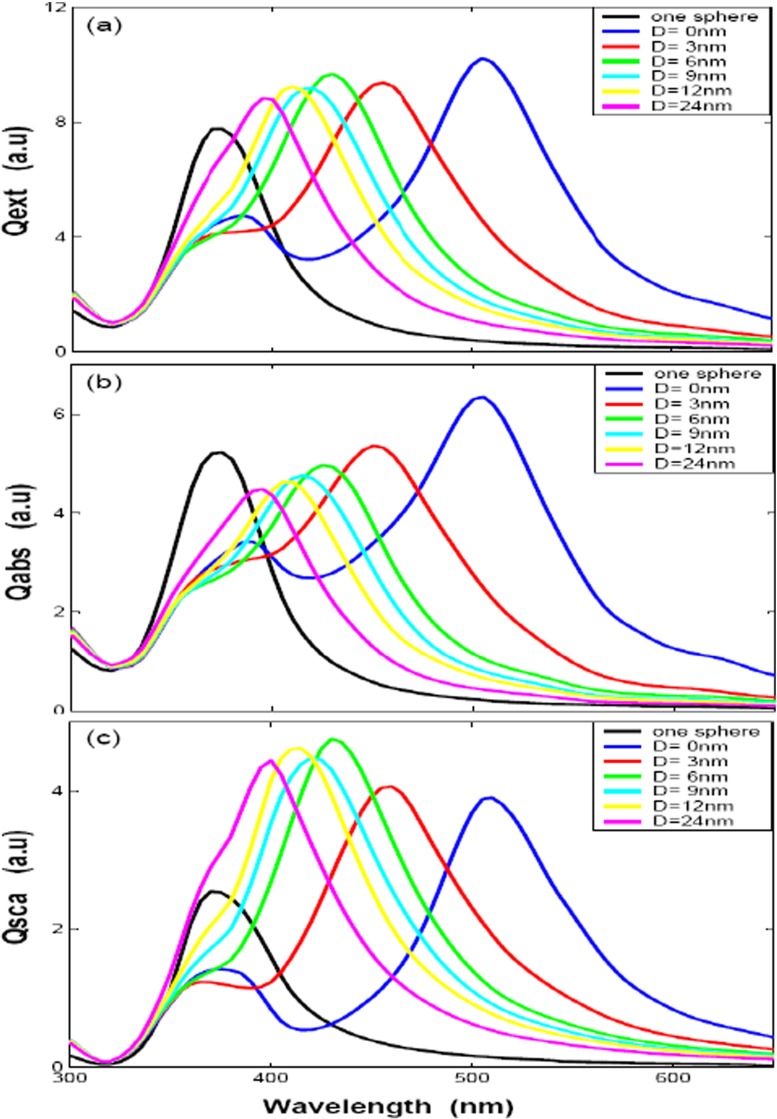
The optical efficiencies of *two spheres* with different gap distances (*D* = 0, 3, 6, 9, 12, and 24 nm) by y direction polarization

In order to get a better understanding about the influences of polarizations to the optical efficiencies with different distances for two silver nanospheres (case of Fig. 1a), the optical efficiencies by two polarizations (**k** along *x* axis), one along the *z* direction and the other *y* direction, are given in Figs. 3a–c and 4a–c, respectively. Figures 3a and 4a show extinction efficiency for two nanosphere with 30 nm radius by *z* polarization and *y* polarization. Compared with each other, we can find out that there is only one peak at about 370 nm by *z* polarization and the peak is slight blue-shifted with big distance. Figure 3a shows the peak of extinction efficiency with different gap distance is almost the same as the single particle case, while the intensity is smaller than the single particle case. Figure 3b, c shows the absorption and scattering efficiencies, we can find out that the intensity of absorption efficiencies is smaller than the single silver sphere while the scattering efficiencies is bigger than the single silver sphere due to augmenting scattering volume, but the absorption efficiency plays the primary contributor to the extinction efficiency. A very different behavior is observed for the other *y* polarization direction, as illustrated in Fig. 4a, there are two peaks by *y* polarization and each spectrum has a common peak at about 370 nm, it means that the plasmon resonance peak of 370 nm is depending on its geometry (silver sphere); the other plasmon resonance peak is obvious red-shift with small distance, what we found that is the dipole resonance starts out about 370 nm, then moves to 400 nm for $\mathit {D}=24$ nm and to 510 nm for $\mathit {D}=0$ nm. The intensity of LSPR extinction efficiency decrease dramatically with increasing the gap distance, which denotes that the interaction strength between the silver nanospheres decays exponentially over the adjacent gap [28], and the absorption and scattering efficiencies show the same properties, the extinction peak red-shifted with big distance in Fig. 4b, c. These properties are similar to gold nanoparticles with DDA simulations and some experiments [29, 30]. It shows that the plasmon resonance strongly red-shifts as the interparticle gap is decreased by polarization parallel to nanosphere axis. One the other hand, there is a very slight blue-shift with decreasing gap for polarization perpendicularity to nanosphere axis. The LSPR shift results from the electromagnetic coupling of the single-particle plasmons, the polarization dependence of which could be explained on the basis of a simple dipole-dipole coupling model. The interparticle interaction is strongly attractive for parallel polarization, which results in the reduction of the plasmon frequency (red-shift of the plasmon band) or the discontinuity of the electrical field normal component is equal to the surface charge density for the boundary conditions of the Maxwell’s equations [31], while the blue-shift for the perpendicular polarization is due to a very weak repulsive interaction between the electronic dipoles of silver nanospheres in the side-by-side case, resulting in increasing the plasmon frequency (blue-shift) or the tangential component of the electrical field across the surface is continuous for the boundary conditions of the Maxwell’s equations. The interparticle interactions are clearly stronger for parallel polarization, as seen from the larger wavelength shifts. These results mean that two silver spheres get “hot spots” only when the wave polarization direction is parallel to the intersphere axis, which agree well with the results of Nie’s experiment [1]. The plasmon resonance changes with the spheres spacing. When a second particle is present in the setup, the LSPR is slightly red-shifted relative to the resonance of a single particle. Increasing the separation distance decreases resonance intensity, blue-shifted the second plasmon peak and width narrows. When the separation become big enough, for example, equal to sphere diameter, the spectra almost overlap with that of a single sphere.

**Fig. 5:**
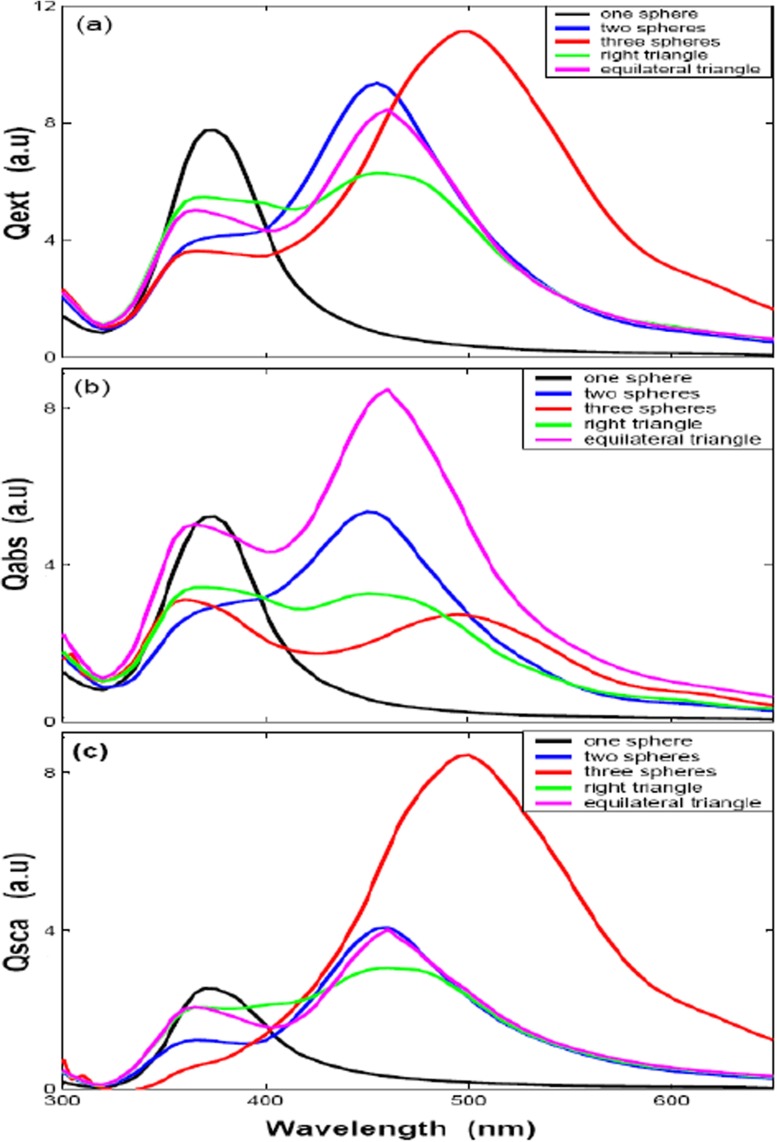
The optical efficiencies of sphere cluster with different shapes *one sphere*, *two sphere*, *three sphere*, and *triangle* (*D* = 6 nm) by *y* direction polarization

Next, we study the influences of nanospheres clusters illuminated by the propagating *x* direction and the electric field *y* polarization of incident plane waves with different shapes to LSPR peak position. The clusters of identical silver nanosphere clusters for two aligned nanospheres, three aligned nanospheres, right triangle, and equilateral triangle with radius 30 nm and 3 nm gap distance to each other are studied, respectively. The optical efficiencies (i.e., extinction efficiency, absorption efficiency, and scattering efficiency) are given in Fig. 5a–c. It shows that when a second/more particle is present in the setup, the LSPR is slightly red-shifted relative to the resonance of a single particle and we also note a significant increase in plasmon width which comes from enhanced radiation damping due to the larger volume of the three identical nanosphere clusters. In addition, the optical properties of the three identical nanospheres with different shapes are also shown clearly in Fig. 5. It shows that the three aligned triangle cluster is the strongest red-shifted compared with right triangle and equilateral triangle nanospheres, whose resonance intensity is bigger than right triangle and equilateral triangle, and peak width is about twice than them. On the other hand, the LSPR peak of the right triangle and equilateral triangle are similar to each other, but the resonance intensity of equilateral triangle is bigger than right triangle mode. The absorption efficiency and scattering efficiency are also given in Fig. 5b, c. Figure 5b shows that the equilateral triangle has the best absorption efficiency than any other modes, and the same to its resonance intensity. Figure 5c shows that scattering efficiencies are similar to each other except three aligned nanospheres. Thus, we could get the results that the three aligned nanospheres has the strongest red-shifts, while the equilateral triangle has the best absorption efficiency and resonance intensity.

**Fig. 6:**
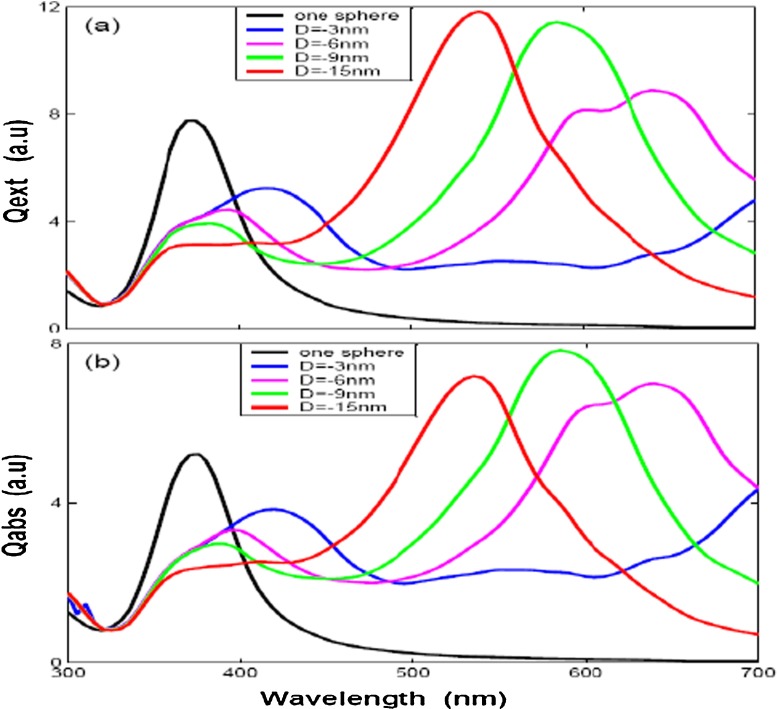
The optical efficiencies of *two spheres* with different gap distances (*D* = −3, −6, −9, and −15 nm) by *y* direction polarization

**Fig. 7:**
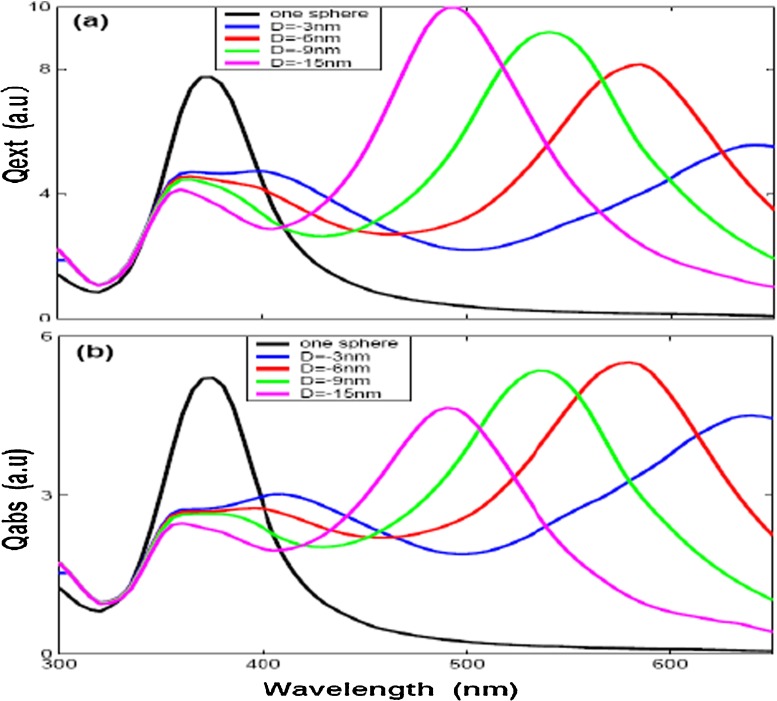
The optical efficiencies of *three spheres* with different gap distances (*D* = −3, −6, −9, and −15 nm) by *y* direction polarization

Fourthly, the optical efficiencies of two aligned nanospheres and equilateral triangle with three identical nanospheres are also investigated, seen in Fig. 1a, c and the simulation results are given in Figs. 6 and 7. The wave propagating direction parallel to the *x* direction and polarization to *y* direction. Where *D* denotes the distance between the adjacent silver nanospheres, and *D* set by negative distance means that we have overlapped the silver nanospheres. Figures 6 and 7 show the similar properties of optical efficiencies for two modes. The results show that the LSPR is obvious red-shift with bigger *D* (the dipole resonance of two overlapped silver nanosphere moves to 520 nm for *D* = −15 nm and to 650 nm for *D* = −6 nm, while the three overlapped silver nanosphere moves to 480 nm for *D* = −15 nm and to 650 nm for *D* = −3 nm) and the resonance intensity of extinction efficiency is increased with bigger *D*, while the peak width is narrowed. On the other hand, the absorption efficiency show the same properties (red-shift with bigger *D*) as extinction efficiency except resonance intensity is decreased with bigger *D*.

**Fig. 8:**
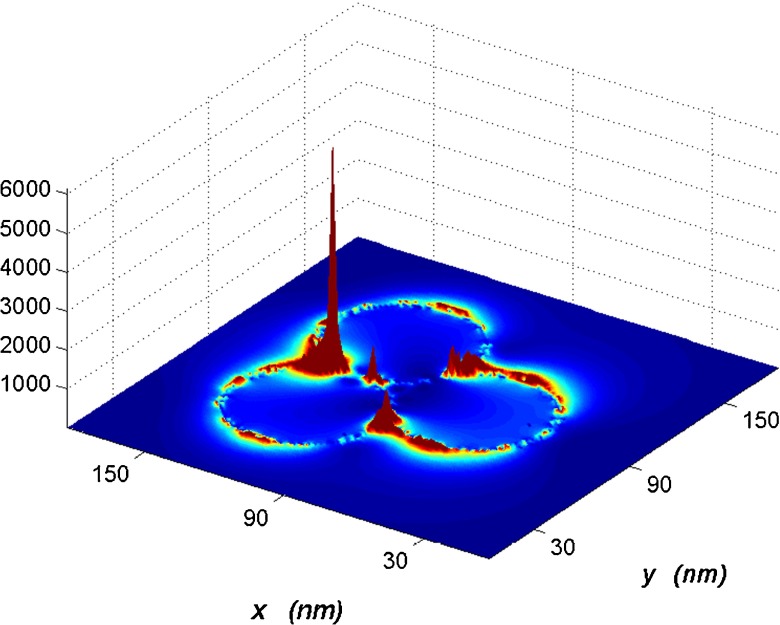
The 3D total electrical field intensity distribution for three touched silver nanospheres ($|\textbf {E}^{2}|$)

**Fig. 9:**
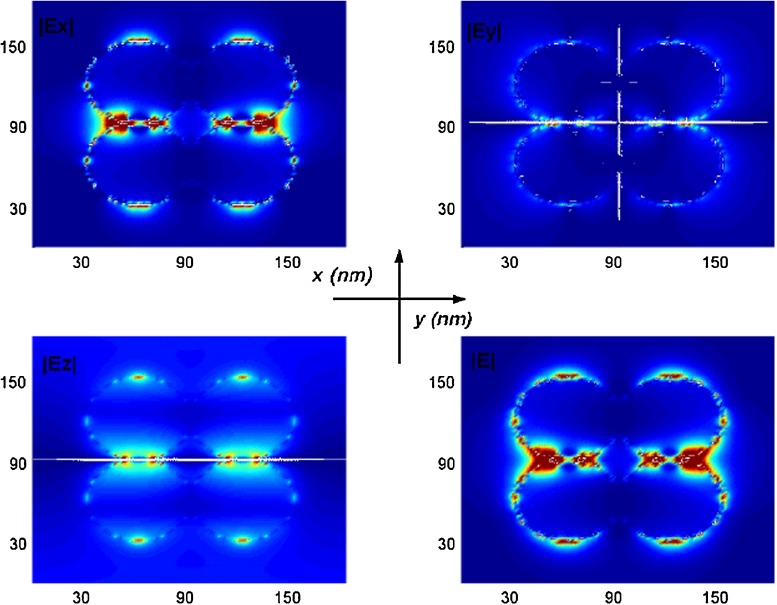
Contour of each electrical field intensity components distribution for four touched silver nanospheres ($|\textbf {E}x|$, $|\textbf {E}y|$, $|\textbf {E}z|$ and total $|\textbf {E}|$)

At last, the distribution of total electrical field intensity for three and four touched spheres illuminated with LSPR wavelength (see Fig. 1 case of c,d) with polarization mode parallel to *x* axis direction are presented in Figs. 8 and 9. The 3D total electric intensity ($|\textbf {E}^{2}|$) of three touched spheres is shown in Fig. 8. It is clear to find that the highest symmetry of electrical field intensity and the electrical field in the central gap differs clearly from those in other gaps; it also shows that there is a highest electrical field enhancement or called “hot spot” within the central gap region, the largest field intensity ($|\textbf {E}^{2}|$) for three touched silver nanospheres resonance are about 6,000 times than the incident field. These enhancements are much larger than studied for single silver nanosphere where factor of 100 enhancement [7]. The enhanced intensity in the central gap are due to the surface plasmon resonance and the concentration of energy flow. It is worth noting that the electrical field intensity of local field in the gaps is quite sensitive to the gap distances; the intensities of electrical field reduce monotonically as the separation distance becomes larger, becoming extremely small at large interparticle distances. In order to get a more detailed discussion on the distributions of electrical field intensity, the distributions of each electrical field intensity components ($|\textbf {E}x|$, $|\textbf {E}y|$, $|\textbf {E}z|$ and total $|\textbf {E}|$) will be given for four touched identical nanospheres in Fig. 9. We can see clearly the intensity associated with the total electric field is well confined on the contact corners. The stronger intensity due to adding more sharp corners or singularities [32], which could be used to broaden the scope of Raman spectroscopy and nanooptics applications. The most significant component of the plasmon intensity field associated with the *x* component corresponds to the incident electric field ($|\textbf {E}x|$) which is major compared to the *y* and *z* components ($|\textbf {E}y|$ and $|\textbf {E}z|$). The reason which could be explained on the boundary conditions of Maxwell’s equations is discontinuity of the electric field component normal to the surface being proportional to polarized surface charge density, while the tangential component of the electrical field across the surface is continuous [31].

## Conclusion

In conclusion, this paper presents studies on two, three, and more silver nanosphere clusters’ LSPR and the distribution of electric field intensity. The intensity could be significantly enhanced due to adding more sharp corners or singularities for small spacing to get more “hot spots” and *x* component of the electric field is major compared to the others. We also find out that “hot spots” are obtained only when the wave polarization direction is parallel to the intersphere axis compared with other polarization directions. The clusters show new types of the local surface plasmon resonances; compared with single silver nanosphere, equilateral triangle nanospheres has a good absorption peak, while the better red-shifted with three aligned nanospheres. The intensity associated with the total electric field is well confined on the contact corners. The manufacturing of novel metal nanoparticles and synthesis of new structures have opened new field for studying material science, e.g., environmental monitoring, Raman scattering and optics, etc. However, development in this field is still in the beginning stage, with many problems waiting to be solved. More broad applications in related fields have yet to happen to meet the demand of social and scientific development. Preparation of uniform and monodisperse materials is critical to the application of surface plasmon resonance. Because scientific research in the field has just begun, many topics have yet to be explored and further studied.
